# Three Three-Axis IEPE Accelerometers on the Inner Liner of a Tire for Finding the Tire-Road Friction Potential Indicators †

**DOI:** 10.3390/s150819251

**Published:** 2015-08-05

**Authors:** Arto Niskanen, Ari J. Tuononen

**Affiliations:** School of Engineering, Aalto University, P.O. Box 14300, Espoo 00076, Finland; E-Mail: ari.tuononen@aalto.fi

**Keywords:** accelerometer, IEPE, intelligent tire, friction, vehicle

## Abstract

Direct tire-road contact friction estimation is essential for future autonomous cars and active safety systems. Friction estimation methods have been proposed earlier for driving conditions in the presence of a slip angle or slip ratio. However, the estimation of the friction from a freely-rolling tire is still an unsolved topic. Knowing the existing friction potential would be beneficial since vehicle control systems could be adjusted before any remarkable tire force has been produced. Since accelerometers are well-known and robust, and thus a promising sensor type for intelligent tires, this study uses three three-axis IEPE accelerometers on the inner liner of a tire to detect friction potential indicators on two equally smooth surfaces with different friction levels. The equal roughness was chosen for both surfaces in order to study the friction phenomena by neglecting the effect of surface texture on vibrations. The acceleration data before the contact is used to differentiate the two friction levels between the tire and the road. In addition, the contact lengths from the three accelerometers are used to validate the acceleration data. A method to differentiate the friction levels on the basis of the acceleration signal is also introduced.

## 1. Introduction

Safe transportation is one of the main goals in vehicle-related research. Current Electronic Stability Control (ESC) and active safety systems, such as Adaptive Cruise Control (ACC) and Automatic Emergency Braking (AEB), are relatively effective and they have increased traffic safety significantly [1]. The active safety and comfort systems have introduced a large sensor network into road vehicles, including several inertial sensors to identify crashes and to estimate actual state of the vehicle. The most important estimated states are yaw rate (direct measurement) and vehicle slip angle (estimated). However, the systems still lack direct tire-road contact information, such as the maximum friction coefficient between the tire and the road. If the friction could be estimated directly in the contact patch, the active safety systems could adapt to different friction situations by optimizing the parameters of the brake system and adjusting the appropriate safety gap for AEB and ACC, for example. In addition, the on-board tire model of the vehicle slip angle estimator could be adjusted for different friction conditions to improve stability control performance even further. The friction estimation is the “killer application” that would enable the breakthrough of tire sensors into the production cars.

The lack of direct contact patch information has led to research on tire sensors. A wide variety of sensors has been used to measure and predict different kinds of phenomena in tire-road contact. Basically, it is possible to measure wheel or suspension part deflections [2,3], ball bearing deflections [4], and tire sidewall deflection [5] to refine valuable information about the tire operating state. However, the sensors installed near the contact patch, such as the tread sensor [6,7] or inner liner accelerometer [8,9], definitely provide more detailed and prompt information about local frictional condition between the tire and the road. Accelerometers have been found to be robust and they are feasible as production tire sensors in the near future. Since the Tire Pressure Monitoring System (TPMS) is already mandatory in new vehicles, tire sensors are no longer only research and development tools. Acceleration signals have been used previously to examine the forces [9] and the progress of aquaplaning in the contact patch [10,11,12] and to determine the road surface conditions [8,13,14]. An accelerometer has also been used to study the energy-harvesting needs for an intelligent tire [15], which is one of the issues to be solved when considering tire sensors.

In the presence of a slip angle or slip ratio, friction and force estimation methods have been proposed [16,17,18,19,20,21]. In these studies, inertial sensor (e.g., accelerometer) measurements have been used to estimate tire-road contact friction and forces directly from the tire [16,17,18], or indirectly from the vehicle [20,21]. However, the estimation of the friction potential from a freely rolling tire is still an unsolved topic. The friction potential is the maximum usable friction in the tire-road contact. Most of the time, the tire is rolling without a considerable slip angle or slip ratio. Knowing the usable friction in these cases might prevent the need for extreme driving maneuvers (e.g., late ESC or AEB intervention) to prevent collisions. This would be beneficial for traffic safety and essential for the autonomous cars of the future. Additionally, the increasing amount of intelligent infrastructure could utilize the friction information produced by the intelligent tires. Road maintenance could be optimized when real-time information from the road is available and information sent from vehicle to vehicle could warn the next road user in slippery conditions.

In case of a freely-rolling tire, the absolute friction value might be impossible to estimate and, thus, the friction potential could be understood as a level of usable friction e.g., low or high friction. Even when the tire is operating in the low-slip range, the maximum friction can be exceeded locally in the contact area due to the non-zero contact stresses. Finding the indicators that link the local sliding of tread elements with the friction potential is one of the main objectives of this study. A model-based estimation of the friction potential from the freely-rolling tire could be possible in the future, however, the indicators of the friction phenomena must be detected first.

In this study the measurements were performed on equally smooth concrete and ice surfaces. The smooth concrete surface was chosen instead of a typical asphalt road in order to eliminate the effect of the surface roughness on the acceleration signals *i.e.*, the pure friction phenomena could be researched. Three three-axis accelerometers were chosen as tire sensors to examine the usability of the well-known sensor type, which has potential to be used in the series production of intelligent tires.

## 2. Experimental Section

### 2.1. Accelerometers

The tire sensors used in this research were three-axis integrated electronic piezoelectric (IEPE) Endevco 35A accelerometers. The measurement range for the acceleration was ±1000 G, which has been experimentally proven to cover the possible accelerations due to the tire deformation at normal driving speeds. The amplitude response is within ±1 dB for the frequency range of 1 Hz to 12 kHz. The maximum transverse sensitivity of the sensors is 5% and, thus, the measurements from all three axes can be considered as independent for the interpretation of the data in this application. The size of the accelerometers was 6.35 × 6.35 × 7.62 mm and their weight was 1.1 gram [22]. The size of the tire sensors should always be considered and they should not affect the properties of the tire, such as its stiffness and the inertia of the contact patch and the carcass. Such small sensors as those used in the research should not have a significant effect on the behavior of the tire.

**Figure 1:**
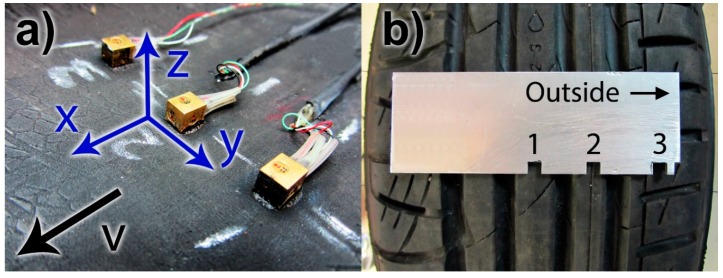
(**a**) Accelerometers attached to the inner liner of the tire; (**b**) Accelerometer positions in the tire (accelerometers attached to the inside).

As shown in Figure 1a, the accelerometers were attached to the inner liner (inner surface) of the tire with cyanoacrylate adhesive. When the accelerometer on the inner liner is against the road surface, the longitudinal direction is the *x*-axis, the lateral direction is the *y*-axis, and the vertical direction is the *z*-axis. In the tire coordinate system, the *x*-axis measures circumferential (tangential), the *y*-axis lateral, and the *z*-axis radial acceleration. The accelerometer coordinate system is shown in Figure 1a.

The accelerometers were positioned side-by-side to cover half of the contact area in the lateral direction as illustrated in Figure 1b. The positions were selected in such a way that the accelerometers were placed behind the contact patch ribs, except the accelerometer 3, which was behind a separate tread block. The attaching of the sensors behind solid tread rubber instead of the grooves allowed the accelerometers to indicate the direct rubber-road contact. With three accelerometers, the different positioning of the sensors could be evaluated and the differences between the solid rib and separate tread block vibrations compared. By mounting the accelerometers side by side, the contact patch shape could also be detected.

### 2.2. Measurements

The experimental data for this research was collected with an instrumented passenger car (Volkswagen Golf V) equipped with an instrumented passenger car summer tire (205/55R16). The tire was fitted with the three three-axis accelerometers introduced above. A slip ring was used to transmit the accelerometer data from the tire to the data acquisition unit and to supply current for the internal preamplifiers of the accelerometers. The slip ring also had an encoder which provided the rotational speed and position of the wheel. Data acquisition was handled with NI DAQ chassis which included an amplifier for the IEPE accelerometers, analog and digital inputs for the different signals, and also a CAN-module to read the optical speed sensor. A sampling rate of 25.6 kHz was used, which gives a spatial resolution of ~0.3 mm between samples at a driving velocity of 30 km/h. The driving velocity was measured with an optical speed sensor.

**Figure 2:**
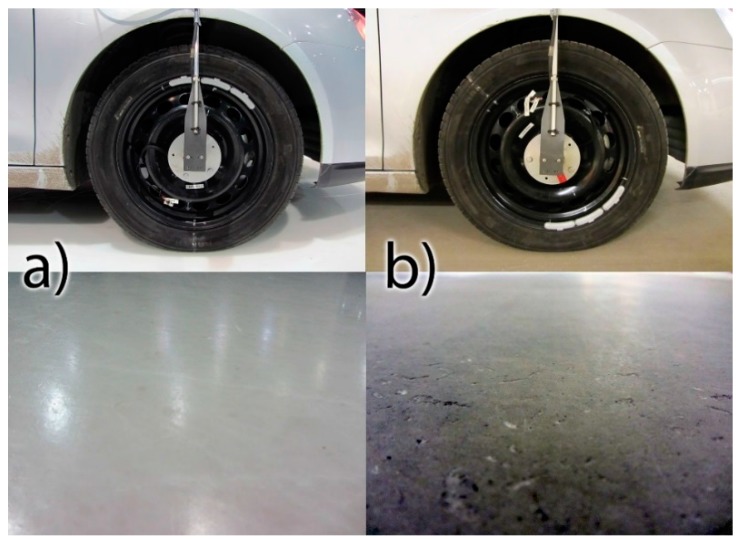
(**a**) Low friction ice surface on the left; (**b**) high friction concrete surface on the right.

Two different surfaces were used to compare low friction and high friction conditions. These surfaces were smooth ice (Figure 2a) and smooth concrete (Figure 2b). The macroscopic surface roughness was similar in both cases, which eliminates the acceleration signal variation resulting from the surface texture and thus the possible pure friction phenomena could be more easily determined from the signal. In the event of a comparison between ice and asphalt, it would be difficult to state if certain vibrations were caused by friction or road roughness. Thus, a macroscopically smooth high-friction concrete surface was selected for this study.

The measurement routine was identical on both surfaces. The car was accelerated to the measurement velocity and the clutch was disengaged before the data logging. The data logging was stopped after several tire rotations. The data that was collected thus presents the freely-rolling tire without any applied driving or braking torque or steering. Three different driving velocities and three different tire pressures were used. The velocities were 10, 20, and 30 km/h and the tire pressures were 2.2, 2.4, and 2.6 bar.

## 3. Results and Discussion

### 3.1. Contact Lengths

**Figure 3:**
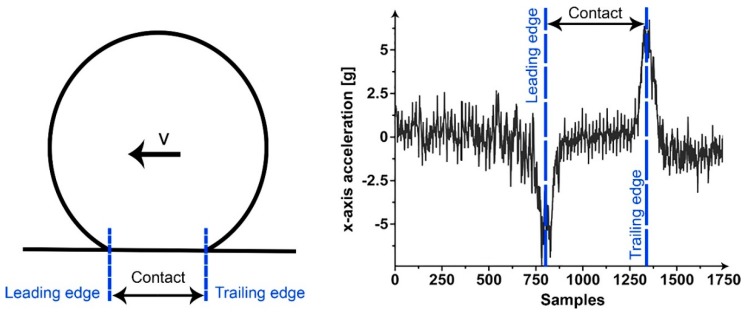
The leading and trailing edge positions are clarified in (**a**); (**b**) Typical *x*-axis acceleration signal at 30 km/h and the leading and trailing edges of the tire-road contact.

Tire-road contact length measurements were performed in the previous research to determine the aquaplaning progress in the contact area [10,11,12]. The same concept was applied in this research. The contact length is the distance between the two peaks in the longitudinal *x*-axis acceleration, which occur at the leading and trailing edges due to the tire deformation. The distance is the time between the peaks (the number of samples divided by the sampling rate) times the accelerometer velocity. The accelerometer velocity is approximated to be the same as the driving velocity. For the freely-rolling tire, the zero wheel slip assumption is valid and the driving velocity can be used to present the velocity of the accelerometers. For the slip conditions, another approach would be necessary, such as the estimation of the dynamic rolling radius, as in [11]. In the Figure 3, a typical longitudinal *x*-axis acceleration signal is plotted and the assumed leading and trailing edges on the corresponding acceleration peaks are marked in the figure. The contact length is, thus, the distance between these two peaks.

**Figure 4:**
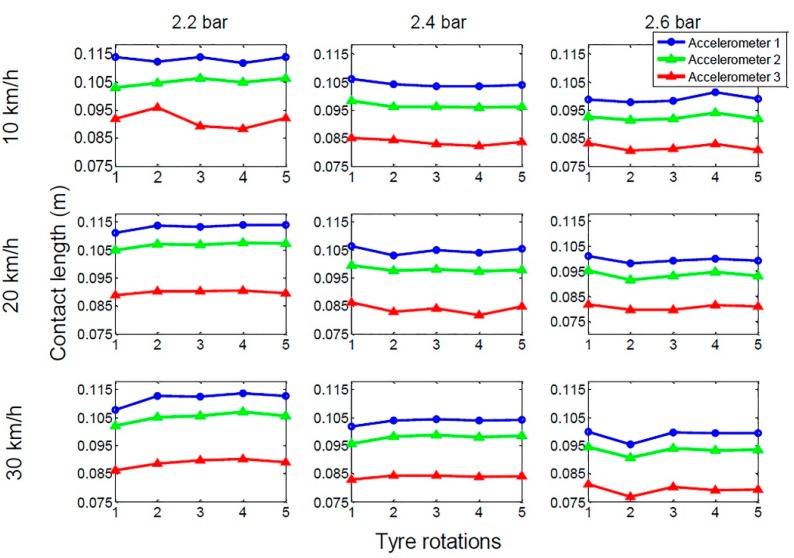
Calculated contact lengths on ice for the three accelerometers. Three different inflation pressures and velocities are shown.

Figure 4 presents the calculated contact lengths on ice for five consecutive tire rotations. The contact lengths are consistent throughout the measurements and, thus, the reliability of the method can be verified. The contact lengths calculated from the data of accelerometer 1, which is located in the center rib, are the longest as a result of the elliptical shape of the contact patch *i.e.*, the contact is longer in the center part of the contact patch than in the shoulder part of the tire. Additionally, the camber angle increases the contact length in the center compared to the outer accelerometers. The second longest contact lengths are measured with accelerometer 2, which is located one rib further than the center one. The shortest contact lengths are in the shoulder part of the tire. The tire pressure has an obvious effect on the contact lengths. The lower the pressure, the longer the contact length. The length of the contact decreases by almost 1.5 cm when the pressure is increased from 2.2 to 2.6 bar.

Contact lengths can also be used to verify the accuracy of the acceleration signal. If the contact length that is acquired is not in the expected range, the signal can be ignored or classified as an outlier and the information can be used in other applications. For example, if the contact length is too long, it can be assumed that the tire pressure is too low and, with the help of TPMS, the vehicle can detect the problem. Or if the camera in the vehicle detects bumps on the road, it can inform the tire sensor about an upcoming disturbance in the acceleration signal. This data can then be ignored during the determination of the contact length.

### 3.2. Friction Potential Indicators

**Figure 5:**
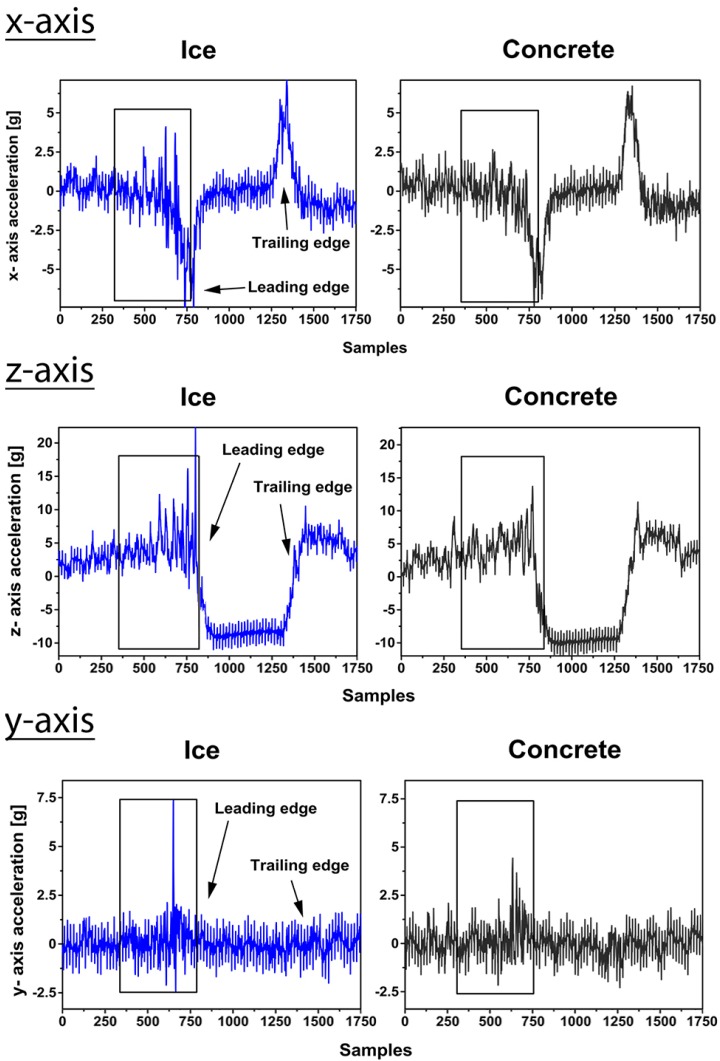
Acceleration signals (*x*, *z*, *y*) from accelerometer 1 on ice and on concrete (2.2 bar, 20 km/h).

For the friction estimation, one of the most interesting parts of the acceleration signal is the section before the contact. In the leading edge, different vibration levels can be seen under different friction conditions as illustrated in Figure 5. In the figure, the signals from all three axes of one accelerometer are shown. With the high friction concrete surface, it is assumed that the acceleration signals which vibrate less are due to the stabilizing effect of the friction between the rubber and the surface. On the ice surface, an increase in the vibration in the leading edge can be seen from the acceleration signals. The lower friction between the rubber and the surface enables the tread blocks to vibrate more as a result of the increased local slip in the contact patch. This phenomenon, however, is rather subtle and is probably detectable only with the equally smooth surfaces.

In the frequency range up to around 1500 Hz, the tire belt and carcass vibrations dominate [23]. These vibrations are governed by the tread band and sidewall properties. Also the vibration caused by the periodic tread block impact against the road fits in this frequency range. The more subtle tread block vibrations are noticeable in higher frequencies. These vibrations are caused by the different vibration modes of the individual tread elements and also the rotational and shear vibrations in tread band as described in [23]. Some experimental studies have been conducted, such as in [24], where a correlation between the tread vibration and tire vibrations was discovered above 1 kHz frequency.

The increase in the vibration on ice is more distinct in the circumferential and radial acceleration than in the lateral *y*-axis acceleration. One reason is that the excitation in the radial and circumferential directions resulting from contact deformation is greater, which can also be recognized from the maximum amplitude of the acceleration signals.

**Figure 6:**
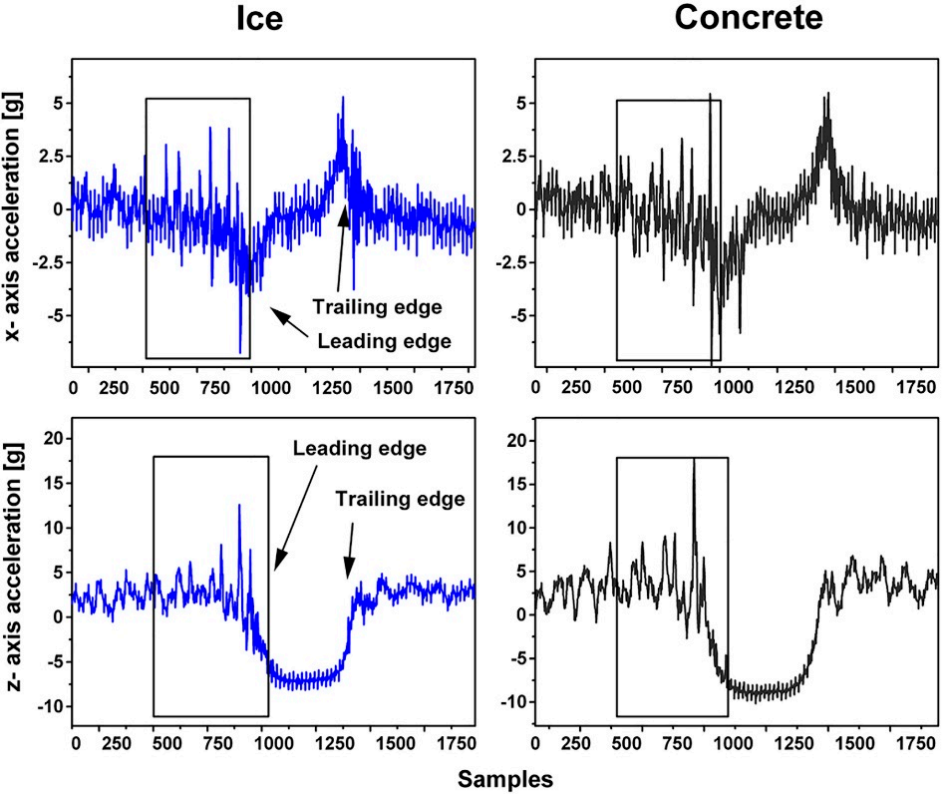
*x*- and *z*-axis acceleration signals from accelerometer 3 on ice and on concrete (2.2 bar, 20 km/h).

Figure 6 presents the *x*- and *z*-axis acceleration signals from accelerometer 3. The vibration level does not vary between the surfaces as much as in the case of accelerometer 1. Accelerometer 3 is placed in the shoulder part of the tire, which contains separate tread blocks. The vibration resulting from the tread blocks hitting the surface obviously covers the friction-related vibration. Accelerometers 1 and 2 were attached behind solid ribs, which do not produce the same kind of vibration as the separate tread blocks. The signal from accelerometer 2 was similar to that from accelerometer 1, but the acceleration levels were lower. Thus, it can be concluded that the best position for the accelerometer is behind a solid rib close to the center of the contact patch.

In order to make the acceleration signal feasible for friction estimation in real-time applications, simple and effective signal processing is required. A method to determine the different friction levels is introduced. By forming a power spectrum, the assumed friction-related vibration was found to exist above 2000 Hz. This is in line with the high frequency range explained earlier. Since the noise is more dominant at the higher frequencies, band-pass filtering was applied to the acceleration signal. An upper limit of 5000 Hz was chosen experimentally to fit the measurements. Tire vibration frequencies are velocity-dependent, but with the measurement velocities in this research (10, 20, and 30 km/h), no significant change in the frequency band needed was found. With higher driving velocities, a moving band-pass filter might be needed. Tread vibration induced by the local slip in the contact, however, can be less velocity-dependent since it consists of tread element vibrations, which are more rubber property-dependent. These properties, of course, change with e.g., varying temperature and aging of the rubber, but should alter the natural frequencies of the tread elements in a limited range.

**Figure 7:**
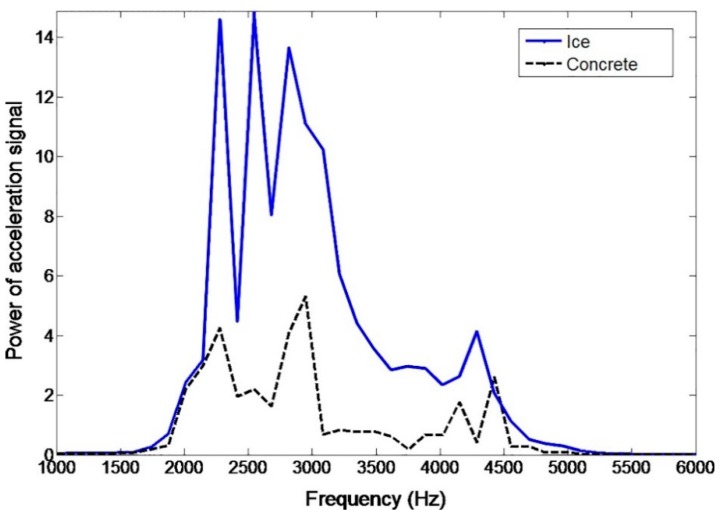
Power spectrum of the band-pass (2000–5000 Hz) filtered *x*-axis acceleration signal before contact (2.2 bar, 20 km/h).

Since accelerometers 2 and 3 do not provide information as useful as that from accelerometer 1, only the information from accelerometer 1 was used in the following results. First, the acceleration data in the leading edge (framed in Figure 5) was band-pass (2000–5000 Hz) filtered and then a Discrete Fourier Transform (DFT) was conducted to find a power spectrum in that frequency range. Figure 7 shows an example of a typical power spectrum for the band-pass filtered *x*-axis acceleration on ice and on concrete. It can be seen that the power spectrum shows larger values due to the assumed friction-related vibration on ice. The area under each power spectrum curve was integrated and the mean value for the area for five tire rotations was calculated. This mean value was then used to compare the different surfaces. It needs to be remarked that the vibration on the ice surface was not significantly larger during every rotation and the phenomenon could be detected by averaging over time.

**Figure 8:**
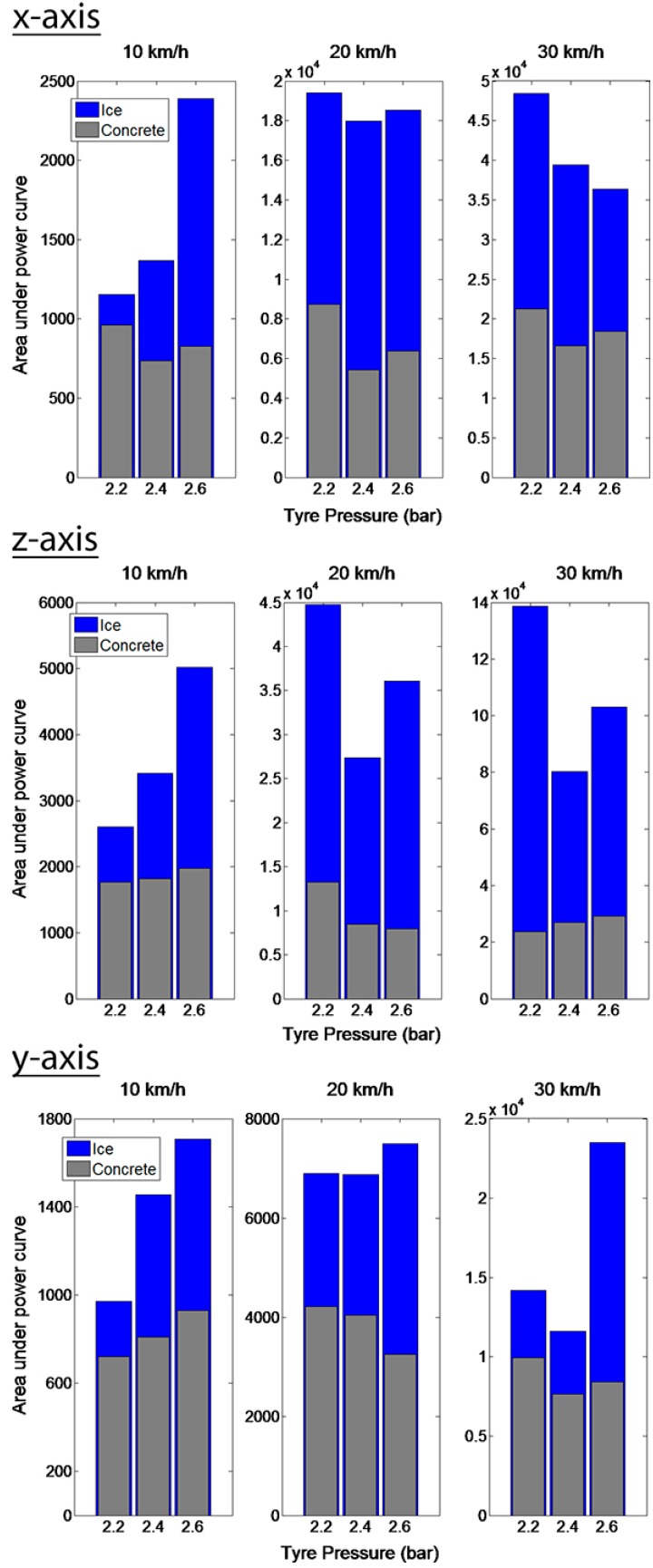
Mean value for the area under the band-pass filtered acceleration (*x*, *z*, *y* from accelerometer 1) power curve on ice (blue) and on concrete (grey) for five consecutive tire rotations.

From Figure 8 it can be seen that the mean value of the area under the power curve is larger in the case of the low-friction ice than in that of the high-friction concrete. The vibration in the tire, resulting from the lower friction level, is greater and, thus, the power curve area is larger. For a velocity of 10 km/h the differences are not as clear as in the case of higher velocities. The area under the power curve of the *y*-axis acceleration does not indicate the friction difference as clearly as the two other axes. That was already discovered from the raw signal shown in Figure 5. For the *x*- and *z*-axes, the area under the power spectrum curve is at least twice as large on ice as on concrete. Interestingly, the radial *z*-axis acceleration provides the most noticeable difference between the two surfaces. The large excitation in the radial direction is one of the reasons, as mentioned earlier.

## 4. Conclusions

Knowing the friction potential in the tire-road contact area would benefit the active safety and stability control systems of vehicles. Friction estimation methods based on the applied slip angle and slip ratio have been proposed earlier, but in the case of a freely-rolling tire, the friction estimation is still an unsolved topic. In this research, three three-axis accelerometers on the inner liner of the tire were used to find friction indicators on smooth ice and concrete surfaces. The surfaces were chosen to have equal roughness to eliminate the effect of the surface texture on the acceleration signals.

The contact lengths calculated from the acceleration signals were used to verify the acceleration data. The contact length information can be very useful for active safety systems in addition to the sensor information that is currently provided in the vehicle. The determination of the contact length was found to be reliable on the two surfaces used in this research.

For friction estimation, the acceleration data before the contact was discovered to contain friction-related information. The vibration level on the lower friction ice surface was higher. It is assumed that the local slip in the leading edge enables the tire carcass to vibrate before contact. On the higher friction concrete surface the vibration level was significantly lower. The friction between the rubber and the surface stabilizes the vibration in the tire carcass. The friction, itself, is a dissipative process as well, and, thus, it is reasonable that a high-friction surface dissipates tire vibrations more than a low-friction one (under similar macro-roughness conditions).

The radial *z-*axis and circumferential *x-*axis accelerations provided the greatest difference in vibration between the two friction levels. The lateral *y-*axis acceleration did not contain as great a difference in vibration levels as the other two. This is due to the lower excitation in the lateral direction. The use of three accelerometers did not offer any benefit for friction-related vibration measurement. The vibration from the separate tread blocks hitting the surface was found to cover the vibration caused by the local slip in the leading edge and, thus, the accelerometer should be positioned behind a solid rib.

As a result of the higher vibration level on a low-friction surface, the area under the acceleration power spectrum curve was larger. The use of the area under the power curve was found to be an effective way to classify the two different friction levels. This could also be implemented in a real-time friction estimation algorithm to evaluate the method on different surfaces with various friction levels. It can be concluded that the area under the acceleration power curve can be linked to the local sliding in the tire-road contact and could be used to differentiate two equally smooth surfaces with different friction levels.
